# Mathematical Analysis and Clinical Implications of an HIV Model with Adaptive Immunity

**DOI:** 10.1155/2019/7673212

**Published:** 2019-11-16

**Authors:** Jaouad Danane, Karam Allali

**Affiliations:** Laboratory of Mathematics and Applications, Faculty of Sciences and Technologies, University Hassan II of Casablanca, P.O. Box 146, Mohammedia, Morocco

## Abstract

In this paper, a mathematical model describing the human immunodeficiency virus (HIV) pathogenesis with adaptive immune response is presented and studied. The mathematical model includes six nonlinear differential equations describing the interaction between the uninfected cells, the exposed cells, the actively infected cells, the free viruses, and the adaptive immune response. The considered adaptive immunity will be represented by cytotoxic T-lymphocytes cells (CTLs) and antibodies. First, the global stability of the disease-free steady state and the endemic steady states is established depending on the basic reproduction number *R*_0_, the CTL immune response reproduction number *R*_1_^*z*^, the antibody immune response reproduction number *R*_1_^*w*^, the antibody immune competition reproduction number *R*_2_^*w*^, and the CTL immune response competition reproduction number *R*_3_^*z*^. On the other hand, different numerical simulations are performed in order to confirm numerically the stability for each steady state. Moreover, a comparison with some clinical data is conducted and analyzed. Finally, a sensitivity analysis for *R*_0_ is performed in order to check the impact of different input parameters.

## 1. Introduction

The human immunodeficiency virus (HIV) is a virus that gradually weakens the immune system since it targets the principal vital immune cells. It is considered as the main cause for several deadly diseases after the resulting acquired immunodeficiency syndrome (AIDS) is reached. With 36.7 million people living with HIV, 1.8 million people becoming newly infected with HIV, and more than 1 million deaths annually, HIV becomes a major global public health issue [1].

In the last decades, many mathematical models describing HIV dynamics were developed [2–11]. With the three main dynamics compartments that are free viruses, healthy CD4^+^ T cells, and infected CD4^+^ T cells, the first viral dynamics was presented and studied in [2]. Including the exposed cells as a new fourth compartment, a modified HIV viral model was tackled in [8]. More recently, the model describing HIV viral dynamics with another fifth compartment representing the cytotoxic T-lymphocytes (CTL) cells is formulated and studied in [9]. The authors study the global stability of the endemic states and illustrate the numerical simulations in order to show the numerical stability for each problem steady state. Notice that the adaptive immunity has two main arms that are cellular and humoral responses. The first one is mediated by CTL cells that play a crucial role in the infection by killing infected cells, while the second arm is mediated by the antibodies which are proteins that are produced by B cells and are specifically programmed to neutralize the viruses [12]. In this paper, we extend the recent work [9] by incorporating to the model this other main component of the adaptive immune response. The dynamics of the HIV infection model including these two arms of the adaptive immune response will be governed by the following nonlinear system of differential equations:

(1) $$\begin{array}{c} \left\{\begin{array}{c} \dot{x}=\lambda -d_{1}x-\frac{k_{1}xv}{x+v},\\ \dot{s}=\frac{k_{1}xv}{x+v}-d_{2}s-k_{2}s,\\ \dot{y}=k_{2}s-d_{3}y-pyz,\\ \dot{v}=ay-d_{4}v-qvw,\\ \dot{w}=gvw-hw,\\ \dot{z}=cyz-bz. \end{array}\right. \end{array}$$

**Figure 1:**
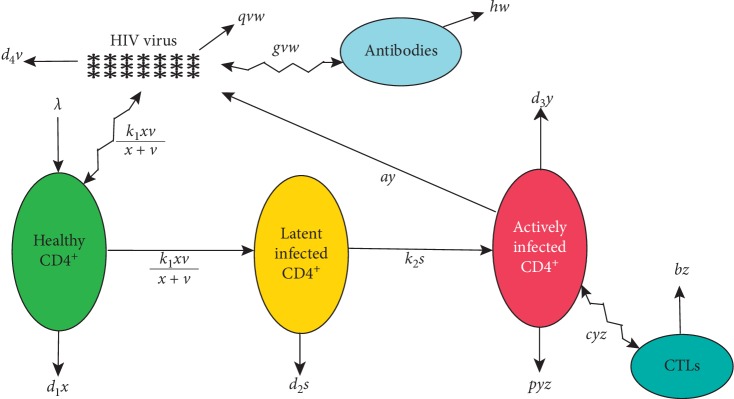
Schematic of the model under consideration.

With the initial conditions, *x*(0)=*x*_0_, *s*(0)=*s*_0_, *y*(0)=*y*_0_, *v*(0)=*v*_0_, *w*(0)=*w*_0_, and *z*(0)=*z*_0_. In this model, *x*, *s*, *y*, *v*, *w*, and *z* denote the concentration of uninfected cells, exposed cells, infected cells, free viruses, antibodies, and CTL cells, respectively. Susceptible host cells, CD4^+^ T cells, are produced at a rate λ and die at a rate *d*_1_*x* and become infected by virus at a rate *k*_1_*xv*/(*x*+*v*). The exposed cells die at a rate (*d*_2_+*k*_2_)*s*. The infected cells increase at rate *k*_2_*s* and decay at rate *d*_3_*y* and are killed by the CTL response at a rate *pyz*. Free viruses are produced by infected cells at a rate *ay* and decay at a rate *d*_4_*v* and are killed by the antibodies at a rate *qvw*. Antibodies develop in response to free viruses at a rate *gvw* and decay at a rate *hw*. Finally, CTLs expand in response to viral antigen derived from infected cells at a rate *cyz* and decay in the absence of antigenic stimulation at a rate *bz*. Note that this model (1), includes the saturated rate, called the saturated mass action [11], which describes better the rate of viral infection. Such HIV viral dynamics is illustrated in Figure 1. The model (1) extends the recent work [9] by adding a new compartment which is the adaptive immune response. In addition to the mathematical analysis of this new model, we will compare our simulations with some clinical data and we will perform a sensitivity analysis of our parameters.

The rest of the paper is organized as follows. The analysis of the model is described in Section 2. In Section 3, we illustrate numerical simulations and compare the model solution to some clinical data. We conclude in the last section.

## 2. Analysis of the Model

### 2.1. Positivity and Boundedness

For the problems dealing with cell population evolution, the cell densities should remain nonnegative and bounded. In this section, we will establish the positivity and boundedness of solutions of the model (1). First of all, for biological reasons, the parameters *x*_0_, *s*_0_, *y*_0_, *v*_0_, *w*_0_, and *z*_0_ must be larger than or equal to 0. Hence, we have the following result:

**Proposition 1 .**

For any initial conditions (*x*_0_, *s*_0_, *y*_0_, *v*_0_, *w*_0_, *z*_0_), system (1) has a unique solution. Moreover, this solution is nonnegative and bounded for all *t* ≥ 0.

**Proof**

By the classical functional differential equations theory (see for instance [13], and the references therein), we can confirm that there is a unique local solution (*x*(*t*), *s*(*t*), *y*(*t*), *v*(*t*), *w*(*t*), *z*(*t*)) to system (1) in [0, *t*_*m*_).

We have the following:

(2) $$\begin{array}{c} {\left.\dot{x}\right|}_{x=0}=\lambda \geq 0,\\ {\left.\dot{s}\right|}_{y=0}=\frac{k_{1}xv}{x+v}\geq 0,\\ {\left.\dot{y}\right|}_{s=0}=k_{2}s\geq 0,\\ {\left.\dot{v}\right|}_{v=0}=ay\geq 0,\\ {\left.\dot{w}\right|}_{w=0}=0\geq 0,\\ {\left.\dot{z}\right|}_{z=0}=0\geq 0, \end{array}$$

this shows the positivity of solutions for *t* ∈ [0, *t*_*m*_). For the boundedness of the solutions,

(3) $$\begin{array}{c} X=x+s+y+\frac{p}{c}z, \end{array}$$

then, we have

(4) $$\begin{array}{c} \dot{X}=\lambda -d_{1}x-d_{2}s-d_{3}y-\frac{bp}{c}z\\ \leq \lambda -\delta X, \end{array}$$

where *δ*=min(*d*_1_, *d*_2_, *d*_3_, *b*). So,

(5) $$\begin{array}{c} X\left(t\right)\leq X\left(0\right)e^{-\delta t}+\frac{\lambda }{\delta }\left(1-e^{-\delta t}\right)\\ \leq X\left(0\right)e^{-\delta t}+\frac{\lambda }{\delta }. \end{array}$$

Similarly, let us consider

(6) $$\begin{array}{c} V=v+\frac{q}{g}w; \end{array}$$

therefore,

(7) $$\begin{array}{c} \dot{V}=ay-d_{4}v-\frac{hq}{g}w\\ \leq ay-\delta V, \end{array}$$

where *α*=min(*d*_4_, *h*), then,

(8) $$\begin{array}{c} V\left(t\right)\leq V\left(0\right)+\frac{a}{\alpha }{\left\|y\right\|}_{\infty }, \end{array}$$

this proves that the solutions *x*(*t*), *s*(*t*), *y*(*t*), *v*(*t*), *w*(*t*), and *z*(*t*) are bounded. Hence, every local solution can be prolonged up to any time *t*_*m*_ > 0, which means that the solution exists globally.

### 2.2. Steady States

System (1) has an infection-free equilibrium *E*_*f*_=(*λ*/*d*_1_, 0,0,0,0,0), corresponding to the maximal level of healthy CD4^+^ T-cells. By simple calculation, the basic reproduction number of (1) is given by

(9) $$\begin{array}{c} R_{0}=\frac{ak_{1}k_{2}}{d_{3}d_{4}\left(d_{2}+k_{2}\right)}=k_{1}\frac{k_{2}}{d_{2}+k_{2}}\times \frac{a}{d_{3}}\times \frac{1}{d_{4}}, \end{array}$$

where *k*_2_/(*d*_2_+*k*_2_) is the proportion of the exposed cells to become productively infected cells, *a*/*d*_3_ is the number of free virus production by an infected cell, and 1/*d*_4_ is the average life of virus. From a biological point of view, *R*_0_ stands for the average number of secondary infections generated by one infected cell when all cells are susceptibles. Depending on the value of this basic reproduction number *R*_0_; in other words, depending on these three biological proportions, we will study the stability of the free-disease and the endemic equilibria. Indeed, it is easy to see that when *R*_0_ > 1, system (1) has four of them. The first endemic equilibrium is *E*_1_=(*x*_1_, *s*_1_, *y*_1_, *v*_1_, *w*_1_, *z*_1_), where

(10) $$\begin{array}{c} x_{1}=\frac{\lambda }{d_{1}+k_{1}\left(1-\left(1/R_{0}\right)\right)},\\ s_{1}=\frac{\lambda k_{1}\left(1-\left(1/R_{0}\right)\right)}{\left(d_{2}+k_{2}\right)\left(d_{1}+k_{1}\left(1-\left(1/R_{0}\right)\right)\right)},\\ y_{1}=\frac{\lambda d_{4}\left(R_{0}-1\right)}{ad_{1}+ak_{1}\left(1-\left(1/R_{0}\right)\right)},\\ v_{1}=\frac{\lambda \left(R_{0}-1\right)}{d_{1}+k_{1}\left(1-\left(1/R_{0}\right)\right)},\\ w_{1}=0,\\ z_{1}=0. \end{array}$$

We define the antibody immune response reproduction number by

(11) $$\begin{array}{c} R_{1}^{w}=\frac{gv_{1}}{h}=\frac{g\lambda \left(R_{0}-1\right)}{d_{1}h+hk_{1}\left(1-\left(1/R_{0}\right)\right)}, \end{array}$$

where 1/*h* is the average life of antibodies cells and *v*_1_ is the number of free viruses at *E*_1_. For the biological significance, *R*_1_^*w*^ represents the average number of the antibodies activated by virus when the viral infection is successful in the absence of CTL immune response.

Furthermore, we introduce the CTL immune response reproduction number given by

(12) $$\begin{array}{c} R_{1}^{z}=\frac{cy_{1}}{b}=\frac{c\lambda d_{4}\left(R_{0}-1\right)}{abd_{1}+abk_{1}\left(1-\left(1/R_{0}\right)\right)}, \end{array}$$

where 1/*b* represents the average life of CTL cells and *y*_1_ is the number of infected cells at *E*_1_. Hence, *R*_1_^*z*^ represents the mean of CTL immune cells activated by an infected cell when the viral infection is successful in the absence of the antibody immune response. The second endemic equilibrium is

(13) $$\begin{array}{c} E_{2}=\left(x_{2},s_{2},y_{2},v_{2},w_{2},z_{2}\right), \end{array}$$

where

(14) $$\begin{array}{c} x_{2}=\frac{-abd_{1}-abk_{1}+\lambda cd_{4}+\sqrt{A}}{2cd_{1}d_{4}},\\ s_{2}=\frac{ak_{1}b\left(-abd_{1}-abk_{1}+\lambda cd_{4}+\sqrt{A}\right)}{cd_{4}\left(d_{2}+k_{2}\right)\left(abd_{1}-abk_{1}+\lambda cd_{4}+\sqrt{A}\right)},\\ y_{2}=\frac{b}{c},\\ v_{2}=\frac{ab}{cd_{4}},\\ w_{2}=0,\\ z_{2}=\frac{d_{2}\left(\left(R_{0}-1\right)\left(-ak_{1}b+\lambda cd_{4}+\sqrt{A}\right)-abd_{1}\left(R_{0}+1\right)\right)}{p\left(abd_{1}-ak_{1}b+\lambda cd_{4}+\sqrt{A}\right)}, \end{array}$$

with *A*=(*abk*_1_ − *λcd*_4_)^2^+*a*^2^*b*^2^*d*_1_^2^+2*a*^2^*b*^2^*d*_1_*k*_1_+2*λabcd*_1_*d*_4_.

We introduce the antibody immune competition reproduction number given by

(15) $$\begin{array}{c} R_{2}^{w}=\frac{gv_{2}}{h}=\frac{abg}{cd_{4}h}, \end{array}$$

with 1/*h* represents the average life of antibodies and *v*_2_ is the number of free viruses at *E*_2_. For biological point of view, *R*_2_^*w*^ represents the average number of the antibodies activated by virus when the viral infection is successful in the absence of CTL response. The third endemic equilibrium is

(16) $$\begin{array}{c} E_{3}=\left(x_{3},s_{3},y_{3},v_{3},w_{3},z_{3}\right), \end{array}$$

where

(17) $$\begin{array}{c} x_{3}=\frac{-d_{1}h-k_{1}h+\lambda g+\sqrt{B}}{2d_{1}g},\\ s_{3}=\frac{k_{1}h\left(-d_{1}h-k_{1}h+\lambda g+\sqrt{B}\right)}{g\left(d_{2}+k_{2}\right)\left(d_{1}h-k_{1}h+\lambda g+\sqrt{B}\right)},\\ y_{3}=\frac{k_{1}k_{2}h\left(-d_{1}h-k_{1}h+\lambda g+\sqrt{B}\right)}{d_{3}g\left(d_{2}+k_{2}\right)\left(d_{1}h-k_{1}h+\lambda g+\sqrt{B}\right)},\\ v_{3}=\frac{h}{g},\\ w_{3}=\frac{d_{4}\left(\left(R_{0}-1\right)\left(-k_{1}h+\lambda g+\sqrt{B}\right)-d_{1}h\left(R_{0}+1\right)\right)}{q\left(d_{1}h-k_{1}h+\lambda g+\sqrt{B}\right)},\\ z_{3}=0, \end{array}$$

with *B*=(*hk*_1_ − *λg*)^2^+*d*_1_^2^*h*^2^+2*k*_1_*d*_1_*h*^2^*k*_1_+2*λgd*_1_*h*.

We define the CTL immune competition reproduction number *R*_3_^*z*^ of our model by

(18) $$\begin{array}{c} R_{3}^{z}=\frac{cy_{3}}{b}=\frac{ck_{1}k_{2}h\left(-d_{1}h-k_{1}h+\lambda g+\sqrt{B}\right)}{bd_{3}g\left(d_{2}+k_{2}\right)\left(d_{1}h-k_{1}h+\lambda g+\sqrt{B}\right)}, \end{array}$$

with 1/*b* represents the average life of CTL cells and *y*_3_ is the number of infected cells at *E*_3_. Hence, *R*_3_^*z*^ represents the average number of CTL immune cells activated by an infected cell when the viral infection is successful in the absence of the antibody immune response. The last endemic equilibrium is

(19) $$\begin{array}{c} E_{4}=\left(x_{4},y_{4},v_{4},w_{4},z_{4}\right), \end{array}$$

where

(20) $$\begin{array}{c} x_{4}=\frac{-d_{1}h-k_{1}h+\lambda g+\sqrt{B}}{2d_{1}g},\\ s_{4}=\frac{k_{1}h\left(-d_{1}h-k_{1}h+\lambda g+\sqrt{B}\right)}{g\left(d_{2}+k_{2}\right)\left(d_{1}h-k_{1}h+\lambda g+\sqrt{B}\right)},\\ y_{4}=\frac{b}{c},\\ v_{4}=\frac{h}{g},\\ w_{4}=\frac{abg-cd_{4}h}{chq}=\frac{d_{4}}{q}\left(R_{2}^{w}-1\right),\\ z_{4}=\frac{\left(ck_{1}k_{2}h-bd_{3}g\left(d_{2}+k_{2}\right)\right)\left(-k_{1}h+\lambda g+\sqrt{B}\right)-d_{1}h\left(ck_{1}k_{2}h+bd_{3}g\left(d_{2}+k_{2}\right)\right)}{pbg\left(d_{2}+k_{2}\right)\left(d_{1}h-k_{1}h+\lambda g+\sqrt{B}\right)},\\ =\frac{d_{3}}{p}\left(R_{3}^{z}-1\right). \end{array}$$

We observe that the second endemic state *E*_2_=(*x*_2_, *y*_2_, *v*_2_, *w*_2_, *z*_2_) exists when *R*_1_^*z*^ > 1. We explain the existence of this endemic equilibrium *E*_2_ as follows. We recall first that, in this state, both the free viruses and CTL cells are present. Assume that *R*_0_ > 1, in the total absence of CTL immune response, the infected cell load per unit time is *λd*_4_(*R*_0_ − 1)/(*ad*_1_+*ak*_1_(1 − (1/*R*_0_))). Via the six equations of model (1), CTL cells are reproduced due to infected cells stimulated per unit time being (*cλd*_4_(*R*_0_ − 1)/(*ad*_1_+*ak*_1_(1 − (1/*R*_0_))))=*cy*_1_. The CTL load during the lifespan of a CTL cell is (*cλd*_4_(*R*_0_ − 1)/(*abd*_1_+*abk*_1_(1 − (1/*R*_0_))))=*R*_1_^*z*^. If (*cλd*_4_(*R*_0_ − 1)/(*abd*_1_+*abk*_1_(1 − (1/*R*_0_)))) > 1, we will have the existence of the endemic equilibrium *E*2. We observe also that the third endemic state *E*_3_=(*x*_3_, *y*_3_, *v*_3_, *w*_3_, *z*_3_) exists when *R*_1_^*w*^ > 1. We explain the existence of this endemic equilibrium *E*_3_ as follows. We recall first that, in this state, both of the free viruses and antibodies are present. Assume that *R*_0_ > 1, in the total absence of the antibody immune response, the viral load per unit time is *λ*(*R*_0_ − 1)/(*d*_1_+*k*_1_(1 − (1/*R*_0_))). Via the six equations of model (1), antibodies are reproduced due to free viruses stimulation per unit time is (*gλ*(*R*_0_ − 1)/(*d*_1_+*k*_1_(1 − (1/*R*_0_))))=*gv*_1_. The viral load during the lifespan of virion is (*gλ*(*R*_0_ − 1)/(*d*_1_*h*+*hk*_1_(1 − (1/*R*_0_))))=*R*_1_^*w*^. If (*gλ*(*R*_0_ − 1)/(*d*_1_*h*+*hk*_1_(1 − (1/*R*_0_)))) > 1, we will have the existence of the endemic equilibrium *E*3. Similarly, one can see that *E*_4_=(*x*_4_, *y*_4_, *v*_4_, *w*_4_, *z*_4_) exists when *R*_3_^*z*^ > 1 and *R*_2_^*w*^ > 1.

### 2.3. Global Stability of the Disease-Free Equilibrium

For the global stability of the disease-free equilibrium, we have the following result.

**Proposition 2 .**

If *R*_0_ ≤ 1, then the endemic point *E*_*f*_ is globally asymptotically stable.

**Proof**

Let the following Lyapunov functional be

(21) $$\begin{array}{c} ℒ\left(x,y,v,w,z\right)=s+\frac{d_{2}+k_{2}}{k_{2}}y+\frac{d_{3}\left(d_{2}+k_{2}\right)}{ak_{2}}v+\frac{q}{g}\frac{d_{3}\left(d_{2}+k_{2}\right)}{ak_{2}}w+\frac{p}{c}\frac{d_{2}+k_{2}}{k_{2}}z. \end{array}$$

The time derivative is given by

(22) $$\begin{array}{c} \dot{ℒ}\left(x,s,y,v,w,z\right)=\dot{s}+\frac{d_{2}+k_{2}}{k_{2}}\dot{y}+\frac{d_{3}\left(d_{2}+k_{2}\right)}{ak_{2}}\dot{v}+\frac{q}{g}\frac{d_{3}\left(d_{2}+k_{2}\right)}{ak_{2}}\dot{w}+\frac{p}{c}\frac{d_{2}+k_{2}}{k_{2}}\dot{z},\\ \dot{ℒ}\left(x,s,y,v,w,z\right)=\frac{k_{1}xv}{x+v}-\left(d_{2}+k_{2}\right)s+\frac{d_{2}+k_{2}}{k_{2}}\left(k_{2}s-d_{3}y\right)-\frac{d_{2}+k_{2}}{k_{2}}pyz\\ +\frac{d_{3}\left(d_{2}+k_{2}\right)}{ak_{2}}\left(ay-d_{4}v-qvw\right)+\frac{q}{g}\frac{d_{3}\left(d_{2}+k_{2}\right)}{ak_{2}}\left(gvw-hw\right)\\ +\frac{p}{c}\frac{d_{2}+k_{2}}{k_{2}}\left(cyz-bz\right),\\ \dot{ℒ}\left(x,s,y,v,w,z\right)=\frac{k_{1}xv}{x+v}-\frac{d_{3}d_{2}}{a}v-\frac{qh}{g}\frac{d_{3}\left(d_{2}+k_{2}\right)}{ak_{2}}w-\frac{bp}{c}\frac{d_{2}+k_{2}}{k_{2}}z,\\ \dot{ℒ}\left(x,y,s,v,w,z\right)\leq k_{1}v-\frac{d_{3}d_{4}\left(d_{2}+k_{2}\right)}{ak_{2}}v\\ \leq \frac{d_{3}d_{4}\left(d_{2}+k_{2}\right)}{ak_{2}}\left(R_{0}-1\right)v. \end{array}$$

If *R*_0_ < 1, then $\dot{ℒ}\leq 0$. Moreover, $\dot{ℒ}\leq 0$ when *v*=0. The largest compact invariant is

(23) $$\begin{array}{c} E=\left\{\left(x,s,y,v,w,z\right)\;|\;v=0\right\}. \end{array}$$

According to LaSalle's invariance principle [14], we have lim_+*∞*_*v*(*t*)=0. The limit system of equations is

(24) $$\begin{array}{c} \left\{\begin{array}{c} \dot{x}=\lambda -d_{1}x,\\ \dot{s}=-d_{2}s-k_{2}s,\\ \dot{y}=k_{2}s-d_{3}y-pyz,\\ \dot{w}=-hw,\\ \dot{z}=cyz-bz. \end{array}\right. \end{array}$$

We define

(25) $$\begin{array}{c} ℒ\left(x,s,y,w,z\right)=\frac{1}{x_{0}}\left(x-x_{0}-x_{0}\ln\frac{x}{x_{0}}\right)+s+\frac{d_{2}+k_{2}}{k_{2}}y+\frac{q}{g}\frac{d_{3}\left(d_{2}+k_{2}\right)}{ak_{2}}w+\frac{d_{2}+k_{2}}{k_{2}}\frac{p}{c}z. \end{array}$$

Since *x*_0_=*λ*/*d*_1_, then

(26) $$\begin{array}{c} \dot{ℒ}\left(x,s,y,w,z\right)=d_{1}\left(2-\frac{x}{x_{0}}-\frac{x_{0}}{x}\right)-\frac{d_{3}\left(d_{2}+k_{2}\right)}{k_{2}}y-\frac{q}{g}\frac{hd_{3}\left(d_{2}+k_{2}\right)}{ak_{2}}w-\frac{d_{2}+k_{2}}{k_{2}}\frac{pb}{c}z. \end{array}$$

Since the arithmetic mean is greater than or equal to the geometric mean, it follows that

(27) $$\begin{array}{c} 2-\frac{x}{x_{0}}-\frac{x_{0}}{x}\leq 0. \end{array}$$

Therefore, $\dot{ℒ}\leq 0$, and the equality holds if *x*=*x*_0_ and *s*=*y*=*w*=*z*=0, which complete the proof.

### 2.4. Global Stability of Infection Steady States

In this subsection, attention is focused on the stability of the infection steady states.

For the first endemic equilibrium *E*_1_, we have the following result.

**Proposition 3 .**

If *R*_0_ > 1, *R*_1_^*z*^ ≤ 1, and *R*_1_^*w*^ ≤ 1, then the endemic point *E*_1_ is globally asymptotically stable.

**Proof**

Let the following Lyapunov functional be

(28) $$\begin{array}{c} ℒ\left(x,s,y,v,w,z\right)=x-x_{1}+\int_{x_{1}}^{x}\frac{\left(d_{2}+k_{2}\right)s_{1}}{k_{1}uv_{1}/\left(u+v_{1}\right)}\text{d}u+s-s_{1}-s_{1}\ln\frac{s}{s_{1}}\\ +\frac{d_{2}+k_{2}}{k_{2}}\left(y-y_{1}-y_{1}\ln\frac{y}{y_{1}}\right)+\frac{d_{3}\left(d_{2}+k_{2}\right)}{ak_{2}}\left(v-v_{1}-v_{1}\ln\frac{v}{v_{1}}\right)\\ +\frac{q}{g}\frac{d_{3}\left(d_{2}+k_{2}\right)}{ak_{2}}w+\frac{p}{c}\frac{d_{2}+k_{2}}{k_{2}}z. \end{array}$$

we have then

(29) $$\begin{array}{c} \dot{ℒ}\left(x,s,y,v,w,z\right)=\dot{x}-\left(d_{2}+k_{2}\right)s_{1}\frac{x+v_{1}}{k_{1}xv_{1}}\dot{x}+\dot{s}-\frac{s_{1}}{s}\dot{s}\\ +\frac{d_{2}+k_{2}}{k_{2}}\left(\dot{y}-\frac{y_{1}}{y}\dot{y}\right)\\ +\frac{d_{3}\left(d_{2}+k_{2}\right)}{ak_{2}}\left(\dot{v}-\frac{v_{1}}{v}\dot{v}\right)\\ +\frac{q}{g}\frac{d_{3}\left(d_{2}+k_{2}\right)}{ak_{2}}\dot{w}+\frac{p}{c}\frac{d_{2}+k_{2}}{k_{2}}\dot{z}. \end{array}$$

On the other hand, we have

(30) $$\begin{array}{c} \left\{\begin{array}{c} \lambda =d_{1}x_{1}+\left(d_{2}+k_{2}\right)s_{1},\\ \frac{k_{1}x_{1}v_{1}}{x_{1}+v_{1}}=\left(d_{2}+k_{2}\right)s_{1},\\ \frac{s_{1}}{v_{1}}=\frac{d_{3}d_{4}}{ak_{2}},\\ \frac{y_{1}}{v_{1}}=\frac{d_{4}}{a},\\ \frac{s_{1}}{y_{1}}=\frac{d_{3}}{k_{2}}. \end{array}\right. \end{array}$$

Hence,

(31) $$\begin{array}{c} \dot{ℒ}\left(x,s,y,v,w,z\right)=\left(\lambda -d_{1}x-\frac{k_{1}xv}{x+v}\right)\left(1-\left(d_{2}+k_{2}\right)s_{1}\frac{x+v_{1}}{k_{1}xv_{1}}\right)\\ +\frac{k_{1}xv}{x+v}-\left(d_{2}+k_{2}\right)s-\frac{s_{1}}{s}\left(\frac{k_{1}xv}{x+v}-\left(d_{2}+k_{2}\right)s\right)\\ +\frac{d_{2}+k_{2}}{k_{2}}\left(k_{2}s-d_{3}y-pyz\right)-\frac{d_{2}+k_{2}}{k_{2}}\frac{y_{1}}{y}\left(k_{2}s-d_{3}y-pyz\right)\\ +\frac{d_{3}\left(d_{2}+k_{2}\right)}{ak_{2}}\left(ay-d_{4}v-qvw\right)-\frac{d_{3}\left(d_{2}+k_{2}\right)}{ak_{2}}\frac{v_{1}}{v}\left(ay-d_{4}v-qvw\right)\\ +\frac{q}{g}\frac{d_{3}\left(d_{2}+k_{2}\right)}{ak_{2}}\left(gvw-hw\right)+\frac{p}{c}\frac{d_{2}+k_{2}}{k_{2}}\left(cyz-bz\right),\\ \dot{ℒ}\left(x,s,y,v,w,z\right)=\lambda -d_{1}x-\left(d_{2}+k_{2}\right)s_{1}\frac{x+v_{1}}{k_{1}xv_{1}}\left(\lambda -d_{1}x-\frac{k_{1}xv}{x+v}\right)\\ -\frac{s_{1}}{s}\left(\frac{k_{1}xv}{x+v}\right)+\left(d_{2}+k_{2}\right)s_{1}+\frac{d_{2}+k_{2}}{k_{2}}py_{1}z+\frac{\left(d_{2}+k_{2}\right)d_{3}}{k_{2}}y_{1}-\left(d_{2}+k_{2}\right)s\frac{y_{1}}{y}-\frac{d_{3}d_{4}\left(d_{2}+k_{2}\right)}{ak_{2}}v\\ +\frac{\left(d_{2}+k_{2}\right)d_{3}}{k_{2}}y_{1}-\left(d_{2}+k_{2}\right)s\frac{y_{1}}{y}-\frac{d_{3}d_{4}\left(d_{2}+k_{2}\right)}{ak_{2}}v-\frac{qh}{g}\frac{d_{3}\left(d_{2}+k_{2}\right)}{ak_{2}}w+\frac{d_{3}\left(d_{2}+k_{2}\right)}{ak_{2}}v_{1}qw-\frac{d_{2}+k_{2}}{k_{2}}\frac{bp}{c}z. \end{array}$$

Thus, this fact implies that

(32) $$\begin{array}{c} \dot{ℒ}\left(x,s,y,v,w,z\right)=\lambda -d_{1}x-\left(d_{2}+k_{2}\right)s_{1}\frac{x+v_{1}}{k_{1}xv_{1}}\left(\lambda -d_{1}x-\frac{k_{1}xv}{x+v}\right)-\frac{s_{1}}{s}\frac{k_{1}xv}{x+v}+d_{2}s_{1}\\ -\frac{d_{3}d_{4}\left(d_{2}+k_{2}\right)}{ak_{2}}v-\frac{d_{3}\left(d_{2}+k_{2}\right)}{k_{2}}\frac{v_{1}}{v}y+\frac{d_{3}\left(d_{2}+k_{2}\right)}{ak_{2}}v_{1}-\frac{qh}{g}\frac{d_{3}\left(d_{2}+k_{2}\right)}{ak_{2}}w+\frac{d_{3}\left(d_{2}+k_{2}\right)}{ak_{2}}qv_{1}w+pz\left(y_{1}-\frac{b}{c}\right). \end{array}$$

Since

(33) $$\begin{array}{c} \left\{\begin{array}{c} \lambda -d_{1}x=d_{1}x_{1}+\left(d_{2}+k_{2}\right)s_{1}-d_{1}x,\\ \lambda -d_{1}x-\left(d_{2}+k_{2}\right)s_{1}\frac{x+v_{1}}{k_{1}xv_{1}}\left(\lambda -d_{1}x-\frac{k_{1}xv}{x+v}\right)=d_{1}x_{1}\left(1-\frac{x}{x_{1}}-\frac{x_{1}}{x}\frac{x+v_{1}}{x_{1}+v_{1}}+\frac{x+v_{1}}{x_{1}+v_{1}}\right)\\ +\left(d_{2}+k_{2}\right)s_{1}\left(1-\frac{x_{1}}{x}\frac{x+v_{1}}{x_{1}+v_{1}}+\frac{v}{v_{1}}\frac{x+v_{1}}{x+v}\right)\\ -\frac{s_{1}}{s}\left(\frac{k_{1}xv}{x+v}\right)+\left(d_{2}+k_{2}\right)s_{1}=-\frac{s_{1}}{s}\frac{xv}{x_{1}v_{1}}\frac{x_{1}+v_{1}}{x+v}\left(d_{2}+k_{2}\right)s_{1}+\left(d_{2}+k_{2}\right)s_{1}, \end{array}\right. \end{array}$$

We have

(34) $$\begin{array}{c} \dot{ℒ}\left(x,s,y,v,w,z\right)=d_{1}x_{1}\left(1-\frac{x}{x_{1}}-\frac{x_{1}}{x}\frac{x+v_{1}}{x_{1}+v_{1}}+\frac{x+v_{1}}{x_{1}+v_{1}}\right)\\ +\left(d_{2}+k_{2}\right)s_{1}\left(1-\frac{x_{1}}{x}\frac{x+v_{1}}{x_{1}+v_{1}}+\frac{v}{v_{1}}\frac{x+v_{1}}{x+v}\right)\\ +\left(d_{2}+k_{2}\right)s_{1}\left(1-\frac{s_{1}}{s}\frac{xv}{x_{1}v_{1}}\frac{x_{1}+v_{1}}{x+v}\right)\\ +\left(d_{2}+k_{2}\right)s_{1}\left(1-\frac{sy_{1}}{s_{1}y}-\frac{v}{v_{1}}\right)\\ +\left(d_{2}+k_{2}\right)s_{1}\left(1-\frac{v_{1}y}{y_{1}v}\right)\\ +\frac{d_{3}\left(d_{2}+k_{2}\right)}{ak_{2}}qw\left(v_{1}-\frac{h}{g}\right)+pz\frac{d_{2}+k_{2}}{k_{2}}\left(y_{1}-\frac{b}{c}\right). \end{array}$$

Therefore,

(35) $$\begin{array}{c} \dot{ℒ}=-\frac{d_{1}v_{1}}{x\left(x_{1}+v_{1}\right)}{\left(x-x_{1}\right)}^{2}\\ +\left(d_{2}+k_{2}\right)s_{1}\left(-1-\frac{v}{v_{1}}+\frac{v}{v_{1}}\frac{x+v_{1}}{x+v}+\frac{x+v}{x+v_{1}}\right)\\ +\left(d_{2}+k_{2}\right)s_{1}\left(5-\frac{x_{1}}{x}\frac{x+v_{1}}{x_{1}+v_{1}}-\frac{s_{1}}{s}\frac{xv}{x_{1}v_{1}}\frac{x_{1}+v_{1}}{x+v}-\frac{sy_{1}}{s_{1}y}-\frac{yv_{1}}{y_{1}v}-\frac{x+v}{x+v_{1}}\right)\\ +\frac{d_{3}\left(d_{2}+k_{2}\right)}{ak_{2}}\frac{h}{g}qw\left(R_{1}^{w}-1\right)+\frac{d_{2}+k_{2}}{k_{2}}\frac{b}{c}pz\left(R_{1}^{z}-1\right), \end{array}$$

which implies that

(36) $$\begin{array}{c} \dot{ℒ}=-\frac{d_{1}v_{1}}{x\left(x_{1}+v_{1}\right)}{\left(x-x_{1}\right)}^{2}\\ -\left(d_{2}+k_{2}\right)s_{1}\left(\frac{x{\left(v-v_{1}\right)}^{2}}{v_{1}\left(x+v_{1}\right)\left(x+v\right)}\right)\\ +\left(d_{2}+k_{2}\right)s_{1}\left(5-\frac{x_{1}}{x}\frac{x+v_{1}}{x_{1}+v_{1}}-\frac{s_{1}}{s}\frac{xv}{x_{1}v_{1}}\frac{x_{1}+v_{1}}{x+v}-\frac{sy_{1}}{s_{1}y}-\frac{yv_{1}}{y_{1}v}-\frac{x+v}{x+v_{1}}\right)\\ +\frac{d_{3}\left(d_{2}+k_{2}\right)}{ak_{2}}\frac{h}{g}qw\left(R_{1}^{w}-1\right)+\frac{d_{2}+k_{2}}{k_{2}}\frac{b}{c}pz\left(R_{1}^{z}-1\right). \end{array}$$

Since the arithmetic mean is greater than or equal to the geometric mean, it follows that

(37) $$\begin{array}{c} 5-\frac{x_{1}}{x}\frac{x+v_{1}}{x_{1}+v_{1}}-\frac{s_{1}}{s}\frac{xv}{x_{1}v_{1}}\frac{x_{1}+v_{1}}{x+v}-\frac{sy_{1}}{s_{1}y}-\frac{yv_{1}}{y_{1}v}-\frac{x+v}{x+v_{1}}\leq 0, \end{array}$$

and we know that *R*_1_^*z*^ < 1 and *R*_1_^*w*^ < 1, then $\dot{L}\leq 0$, and the equality holds when *x*=*x*_1_, *y*=*y*_1_, *v*=*v*_1_, *w*=*w*_1_, and *z*=*z*_1_. By the LaSalle invariance principle [14], the endemic point *E*_1_ is asymptotically stable when *R*_0_ > 1.

For the second endemic equilibrium *E*_2_, we have the following result.

**Proposition 4 .**

If *R*_0_ > 1, *R*_1_^*z*^ > 1, and *R*_2_^*w*^ ≤ 1, then the endemic point *E*_2_ is globally asymptotically stable.

**Proof**

Let the following Lyapunov functional be

(38) $$\begin{array}{c} ℒ\left(x,s,y,v,w,z\right)=x-x_{2}-\int_{x_{2}}^{x}\frac{\left(d_{2}+k_{2}\right)s_{2}}{k_{1}uv_{2}/\left(u+v_{2}\right)}\text{d}u+s-s_{2}-s_{2}\ln\frac{s}{s_{2}}\\ +\frac{d_{2}+k_{2}}{k_{2}}\left(y-y_{2}-y_{2}\ln\frac{y}{y_{2}}\right)+\frac{d_{3}\left(d_{2}+k_{2}\right)+\left(d_{2}+k_{2}\right)pz_{2}}{ak_{2}}\\ \times \left(v-v_{2}-v_{2}\ln\frac{v}{v_{2}}\right)+\frac{q}{g}\frac{d_{3}\left(d_{2}+k_{2}\right)+\left(d_{2}+k_{2}\right)pz_{2}}{ak_{2}}w\\ +\frac{p}{c}\frac{d_{2}+k_{2}}{k_{2}}\left(z-z_{2}-z_{2}\ln\frac{z}{z_{2}}\right), \end{array}$$

then, we have

(39) $$\begin{array}{c} \dot{ℒ}\left(x,y,s,v,w,z\right)=\dot{x}-\left(d_{2}+k_{2}\right)s_{2}\frac{x+v_{2}}{k_{1}xv_{2}}\dot{x}+\dot{s}-\frac{s_{2}}{s}\dot{s}+\frac{d_{2}+k_{2}}{k_{2}}\left(\dot{y}-\frac{y_{2}}{y}\dot{y}\right)\\ +\frac{d_{3}\left(d_{2}+k_{2}\right)+\left(d_{2}+k_{2}\right)pz_{2}}{ak_{2}}\left(\dot{v}-\frac{v_{2}}{v}\dot{v}\right)+\frac{q}{g}\frac{d_{3}\left(d_{2}+k_{2}\right)+\left(d_{2}+k_{2}\right)pz_{2}}{ak_{2}}\dot{w}\\ +\frac{p}{c}\frac{d_{2}+k_{2}}{k_{2}}\left(\dot{z}-\frac{z_{2}}{z}\dot{z}\right). \end{array}$$

We know that

(40) $$\begin{array}{c} \left\{\begin{array}{c} \lambda =d_{1}x_{2}+\left(d_{2}+k_{2}\right)s_{2},\\ k_{1}x_{2}v_{2}=\left(x_{2}+v_{2}\right)\left(d_{2}+k_{2}\right)s_{2},\\ \frac{s_{2}}{y_{2}}=\frac{d_{3}}{k_{2}}+\frac{pz_{2}}{k_{2}},\\ \frac{y_{2}}{v_{2}}=\frac{d_{4}}{a},\\ \frac{s_{2}}{v_{2}}=\frac{d_{3}d_{4}}{ak_{2}}+\frac{d_{4}pz_{2}}{ak_{2}}, \end{array}\right. \end{array}$$

so, we have

(41) $$\begin{array}{c} \dot{ℒ}\left(x,s,y,v,z\right)=\lambda -d_{1}x-\frac{x_{2}}{x}\frac{x+v_{2}}{x_{2}+v_{2}}\left(\lambda -d_{1}x\right)+\left(\left(d_{2}+k_{2}\right)s_{2}\right)\frac{v}{v_{2}}\frac{x_{2}+v_{2}}{x+v}-\left(d_{2}+k_{2}\right)s-\frac{s_{2}}{s}\left(\frac{k_{1}xv}{x+v}-\left(d_{2}+k_{2}\right)s\right)+\frac{d_{2}+k_{2}}{k_{2}}\left(k_{2}s-d_{3}y-pyz\right)-\frac{d_{2}+k_{2}}{k_{2}}\frac{y_{2}}{y}\left(k_{2}s-d_{3}y-pyz\right)+\frac{d_{3}\left(d_{2}+k_{2}\right)+\left(d_{2}+k_{2}\right)pz_{2}}{ak_{2}}\times \left(ay-d_{4}v-qvw\right)-\frac{d_{3}\left(d_{2}+k_{2}\right)+\left(d_{2}+k_{2}\right)pz_{2}}{ak_{2}}\frac{v_{2}}{v}\left(ay-d_{4}v-qvw\right)+\frac{q}{g}\frac{d_{3}\left(d_{2}+k_{2}\right)+\left(d_{2}+k_{2}\right)pz_{2}}{ak_{2}}\left(gvw-hw\right)+\frac{p}{c}\frac{d_{2}+k_{2}}{k_{2}}\left(cyz-bz\right)-\frac{pz_{2}}{cz}\frac{d_{2}+k_{2}}{k_{2}}\left(cyz-bz\right). \end{array}$$

On the other hand, we have

(42) $$\begin{array}{c} \left\{\begin{array}{c} \lambda -d_{1}x=\left(d_{1}x_{2}+d_{2}+k_{2}\right)s_{2}-d_{1}x,\\ \lambda -d_{1}x-\left(d_{2}+k_{2}\right)s_{2}\frac{x+v_{2}}{k_{1}xv_{2}}\left(\lambda -d_{1}x-\frac{k_{1}xv}{x+v}\right)=d_{1}x_{2}\left(1-\frac{x}{x_{2}}-\frac{x_{2}}{x}\frac{x+v_{2}}{x_{2}+v_{2}}+\frac{x+v_{2}}{x_{2}+v2}\right)\\ +\left(d_{2}+k_{2}\right)s_{2}\left(1-\frac{x_{2}}{x}\frac{x+v_{2}}{x_{2}+v_{2}}+\frac{v}{v_{2}}\frac{x+v_{2}}{x+v}\right)\\ -\frac{s_{2}}{s}\left(\frac{k_{1}xv}{x+v}\right)+\left(d_{2}+k_{2}\right)s_{2}=\left(d_{2}+k_{2}\right)s_{2}\left(1-\frac{s_{2}}{s}\frac{xv}{x_{2}v_{2}}\frac{x_{2}+v_{2}}{x+v}\right), \end{array}\right.\\ \left\{\begin{array}{c} \frac{\left(d_{2}+k_{2}\right)d_{3}}{k_{2}}y_{2}-\left(d_{2}+k_{2}\right)s\frac{y_{2}}{y}-\frac{\left(d_{2}+k_{2}\right)d_{3}d_{4}}{ak_{2}}v\\ =\left(d_{2}+k_{2}\right)s_{2}\left(1-\frac{s}{s_{2}}\frac{y_{2}}{y}-\frac{v}{v_{2}}\right)+\frac{\left(d_{2}+k_{2}\right)}{k_{2}}pz_{2}y_{2}\frac{v}{v_{2}}-\frac{\left(d_{2}+k_{2}\right)}{k_{2}}pz_{2}y_{2},\\ \frac{d_{4}d_{3}\left(d_{2}+k_{2}\right)}{ak_{2}}v_{2}-\frac{d_{3}\left(d_{2}+k_{2}\right)}{k_{2}}\frac{v_{2}}{v}y=\left(d_{2}+k_{2}\right)s_{2}\left(1-\frac{y}{y_{2}}\frac{v_{2}}{v}\right)\\ +\frac{\left(d_{2}+k_{2}\right)}{k_{2}}pz_{2}y\frac{v_{2}}{v}-\frac{\left(d_{2}+k_{2}\right)}{k_{2}}pz_{2}y_{2}, \end{array}\right.\\ \left\{\begin{array}{c} \frac{\left(d_{2}+k_{2}\right)pz_{2}}{ak_{2}}\left(ay-d_{4}v\right)-\frac{\left(d_{2}+k_{2}\right)pz_{2}}{ak_{2}}\frac{v_{2}}{v}\left(ay-d_{4}v\right)=\frac{\left(d_{2}+k_{2}\right)pz_{2}y}{k_{2}}\\ -\frac{\left(d_{2}+k_{2}\right)pz_{2}y_{2}}{k_{2}}\frac{v}{v_{2}}-\frac{\left(d_{2}+k_{2}\right)pz_{2}y}{k_{2}}\frac{v_{2}}{v}+\frac{\left(d_{2}+k_{2}\right)pz_{2}y_{2}}{k_{2}},\\ \frac{\left(d_{2}+k_{2}\right)pz_{2}}{ak_{2}}\left(ay-d_{4}v\right)-\frac{\left(d_{2}+k_{2}\right)pz_{2}}{ak_{2}}\frac{v_{2}}{v}\left(ay-d_{4}v\right)-\frac{z_{2}}{z}\frac{p}{c}\frac{d_{2}+k_{2}}{k_{2}}\left(cyz-bz\right)\\ +\frac{\left(d_{2}+k_{2}\right)}{k_{2}}pz_{2}y_{2}\frac{v}{v_{2}}-\frac{\left(d_{2}+k_{2}\right)}{k_{2}}pz_{2}y_{2}+\frac{\left(d_{2}+k_{2}\right)}{k_{2}}pz_{2}y\frac{v_{2}}{v}-\frac{\left(d_{2}+k_{2}\right)}{k_{2}}pz_{2}y_{2}=0. \end{array}\right. \end{array}$$

This fact implies that

(43) $$\begin{array}{c} \dot{L}=-\frac{d_{1}v_{2}}{x\left(x_{2}+v_{2}\right)}{\left(x-x_{2}\right)}^{2}\\ -\left(d_{2}+k_{2}\right)s_{2}\left(\frac{x{\left(v-v_{2}\right)}^{2}}{v_{2}\left(x+v_{2}\right)\left(x+v\right)}\right)\\ +\left(d_{2}+k_{2}\right)s_{2}\left(5-\frac{x_{2}}{x}\frac{x+v_{2}}{x_{2}+v_{2}}-\frac{s_{2}}{s}\frac{xv}{x_{2}v_{2}}\frac{x_{2}+v_{2}}{x+v}-\frac{sy_{2}}{s_{2}y}-\frac{yv_{2}}{y_{2}v}-\frac{x+v}{x+v_{2}}\right)\\ +\frac{h}{g}\frac{d_{3}\left(d_{2}+k_{2}\right)+\left(d_{2}+k_{2}\right)pz_{2}}{ak_{2}}qw\left(R_{2}^{w}-1\right). \end{array}$$

Since the arithmetic mean is greater than or equal to the geometric mean, it follows that

(44) $$\begin{array}{c} 5-\frac{x_{2}}{x}\frac{x+v_{2}}{x_{2}+v_{2}}-\frac{s_{2}}{s}\frac{xv}{x_{2}v_{2}}\frac{x_{2}+v_{2}}{x+v}-\frac{sy_{2}}{s_{2}y}-\frac{yv_{2}}{y_{2}v}-\frac{x+v}{x+v_{2}}\leq 0, \end{array}$$

and we know that *R*_2_^*w*^ < 1 which means that $\dot{L}\leq 0$, and the equality holds when *x*=*x*_2_, *s*=*s*_2_, *y*=*y*_2_, *v*=*v*_2_, *w*=*w*_2_, and *z*=*z*_2_. By the LaSalle invariance principle [14], the endemic point *E*_2_ is asymptotically stable.

For the third endemic equilibrium *E*_3_, we have the following result.

**Proposition 5 .**

If *R*_0_ > 1, *R*_3_^*z*^ ≤ 1, and *R*_1_^*w*^ > 1, then the endemic point *E*_3_ is globally asymptotically stable.

**Proof**

Let the following Lyapunov functional be

(45) $$\begin{array}{c} ℒ\left(x,s,y,v,w,z\right)=x-x_{3}-\int_{x_{3}}^{x}\frac{\left(d_{2}+k_{2}\right)s_{3}}{k_{1}uv_{3}/\left(u+v_{3}\right)}\text{d}u+s-s_{3}-s_{3}\ln\frac{s}{s_{3}}\\ +\frac{d_{2}+k_{2}}{k_{2}}\left(y-y_{3}-y_{3}\ln\frac{y}{y_{3}}\right)+\frac{d_{3}\left(d_{2}+k_{2}\right)}{ak_{2}}\\ \times \left(v-v_{3}-v_{3}\ln\frac{v}{v_{3}}\right)+\frac{d_{3}\left(d_{2}+k_{2}\right)}{ak_{2}}\frac{q}{g}\left(w-w_{3}-w_{3}\ln\frac{w}{w_{3}}\right)\\ +\frac{p}{c}\frac{d_{2}+k_{2}}{k_{2}}z. \end{array}$$

Then, we have

(46) $$\begin{array}{c} \dot{ℒ}\left(x,s,y,v,w,z\right)=\dot{x}-\left(d_{2}+k_{2}\right)s_{3}\frac{x+v_{3}}{k_{1}xv_{3}}\dot{x}+\dot{s}-\frac{s_{3}}{s}\dot{s}+\frac{\left(d_{2}+k_{2}\right)}{k_{2}}\left(\dot{y}-\frac{y_{3}}{y}\dot{y}\right)\\ +\frac{d_{3}\left(d_{2}+k_{2}\right)}{ak_{2}}\left(\dot{v}-\frac{v_{3}}{v}\dot{v}\right)+\frac{d_{3}\left(d_{2}+k_{2}\right)}{ak_{2}}\frac{q}{g}\left(\dot{w}-\frac{w_{3}}{w}\dot{w}\right)+\frac{p}{c}\dot{z}; \end{array}$$

this fact implies that

(47) $$\begin{array}{c} \dot{ℒ}\left(x,s,y,v,w,z\right)=\lambda -d_{1}x-\left(d_{2}+k_{2}\right)s_{3}\frac{x+v_{3}}{k_{1}xv_{3}}\left(\lambda -d_{1}x-\frac{k_{1}xv}{x+v}\right)-\frac{s_{3}}{s}\frac{k_{1}xv}{x+v}+\left(d_{2}+k_{2}\right)s_{3}\\ +\frac{d_{3}\left(d_{2}+k_{2}\right)}{k_{2}}y_{3}-\left(d_{2}+k_{2}\right)s\frac{y_{3}}{y}-\frac{d_{4}d_{3}\left(d_{2}+k_{2}\right)}{ak_{2}}v\\ -\frac{d_{3}\left(d_{2}+k_{2}\right)}{k_{2}}\frac{v_{3}}{v}y+\frac{d_{3}d_{4}\left(d_{2}+k_{2}\right)}{ak_{2}}v_{3}-\frac{d_{3}\left(d_{2}+k_{2}\right)}{ak_{2}}qw_{3}v\\ +\frac{d_{3}\left(d_{2}+k_{2}\right)}{ak_{2}}qv_{3}w_{3}+\frac{\left(d_{2}+k_{2}\right)}{k_{2}}pz\left(y_{3}-\frac{b}{c}\right). \end{array}$$

We know that

(48) $$\begin{array}{c} \left\{\begin{array}{c} \lambda =d_{1}x_{3}+\left(d_{2}+k_{2}\right)s_{3},\\ k_{1}x_{3}v_{3}=\left(x_{3}+v_{3}\right)\left(d_{2}+k_{2}\right)s_{3},\\ \frac{s_{3}}{y_{3}}=\frac{d_{3}}{k_{2}},\\ \frac{y_{3}}{v_{3}}=\frac{d_{4}}{a}+\frac{qw_{3}}{a},\\ \frac{s_{2}}{v_{2}}=\frac{d_{3}d_{4}}{ak_{2}}+\frac{d_{3}qw_{3}}{ak_{2}}. \end{array}\right. \end{array}$$

So, we have

(49) $$\begin{array}{c} \left\{\begin{array}{c} \lambda -d_{1}x=d_{1}x_{3}+\left(d_{2}+k_{2}\right)s_{3}-d_{1}x,\\ \lambda -d_{1}x-\left(d_{2}+k_{2}\right)s_{3}\frac{x+v_{3}}{k_{1}xv_{3}}\left(\lambda -d_{1}x-\frac{k_{1}xv}{x+v}\right)=d_{1}x_{3}\left(1-\frac{x}{x_{3}}-\frac{x_{3}}{x}\frac{x+v_{3}}{x_{3}+v_{3}}+\frac{x+v_{3}}{x_{3}+v_{3}}\right)\\ +\left(d_{2}+k_{2}\right)s_{3}\left(1-\frac{x_{3}}{x}\frac{x+v_{3}}{x_{3}+v_{3}}+\frac{v}{v_{3}}\frac{x+v_{3}}{x+v}\right)\\ -\frac{s_{3}}{s}\left(\frac{k_{1}xv}{x+v}\right)+\left(d_{2}+k_{2}\right)s_{3}=\left(d_{2}+k_{2}\right)s_{3}\left(1-\frac{s_{3}}{s}\frac{xv}{x_{3}v_{3}}\frac{x_{3}+v_{3}}{x+v}\right), \end{array}\right.\\ \left\{\begin{array}{c} \frac{\left(d_{2}+k_{2}\right)d_{3}}{k_{2}}y_{3}-\left(d_{2}+k_{2}\right)s\frac{y_{3}}{y}=\left(d_{2}+k_{2}\right)s_{3}\left(1-\frac{s}{s_{3}}\frac{y_{3}}{y}\right),\\ -\frac{d_{3}d_{4}\left(d_{2}+k_{2}\right)}{ak_{2}}v-\frac{d_{3}\left(d_{2}+k_{2}\right)}{k_{2}}\frac{v_{3}}{v}y+\frac{d_{3}d_{4}\left(d_{2}+k_{2}\right)}{ak_{2}}v_{3}-\frac{d_{3}\left(d_{2}+k_{2}\right)}{ak_{2}}qw_{3}v+\frac{d_{3}\left(d_{2}+k_{2}\right)}{ak_{2}}qv_{3}w_{3}\\ =\left(d_{2}+k_{2}\right)s_{3}\left(1-\frac{v}{v_{3}}-\frac{v_{3}}{v}\frac{y}{y_{3}}\right). \end{array}\right. \end{array}$$

Then, we have

(50) $$\begin{array}{c} \dot{L}=-\frac{d_{1}v_{3}}{x\left(x_{3}+v_{3}\right)}{\left(x-x_{3}\right)}^{2}\\ -\left(d_{2}+k_{2}\right)s_{3}\left(\frac{x{\left(v-v_{3}\right)}^{2}}{v_{3}\left(x+v_{3}\right)\left(x+v\right)}\right)\\ +\left(d_{2}+k_{2}\right)s_{3}\left(5-\frac{x_{3}}{x}\frac{x+v_{3}}{x_{3}+v_{3}}-\frac{s_{3}}{s}\frac{xv}{x_{3}v_{3}}\frac{x_{3}+v_{3}}{x+v}-\frac{sy_{3}}{s_{3}y}-\frac{yv_{3}}{y_{3}v}-\frac{x+v}{x+v_{3}}\right),\\ +\frac{b}{c}\frac{d_{2}+k_{2}}{k_{2}}pz\left(R_{3}^{z}-1\right). \end{array}$$

Since the arithmetic mean is greater than or equal to the geometric mean, it follows that

(51) $$\begin{array}{c} 5-\frac{x_{3}}{x}\frac{x+v_{3}}{x_{3}+v_{3}}-\frac{s_{3}}{s}\frac{xv}{x_{3}v_{3}}\frac{x_{3}+v_{3}}{x+v}-\frac{sy_{3}}{s_{3}y}-\frac{yv_{3}}{y_{3}v}-\frac{x+v}{x+v_{3}}\leq 0, \end{array}$$

and we know that *R*_3_^*z*^ < 1, then $\dot{L}\leq 0$, and the equality holds when *x*=*x*_3_, *s*=*s*_3_, *y*=*y*_3_, *v*=*v*_3_, *w*=*w*_3_, and *z*=*z*_3_. By the LaSalle invariance principle [14], the endemic point *E*_3_ is asymptotically stable when *R*_0_ > 1.

Finally, for the last endemic equilibrium *E*_4_, we have the following result.

**Proposition 6 .**

If *R*_0_ > 1, *R*_3_^*z*^ > 1, and *R*_2_^*w*^ > 1, then the endemic point *E*_4_ is globally asymptotically stable.

**Proof**

Let the following Lyapunov functional be

(52) $$\begin{array}{c} ℒ\left(x,s,y,v,w,z\right)=x-x_{4}-\int_{x_{4}}^{x}\frac{\left(d_{2}+k_{2}\right)s_{4}}{k_{1}uv_{4}/\left(u+v_{4}\right)}\text{d}u+s-s_{4}-s_{4}\ln\frac{s}{s_{4}}\\ +\frac{d_{2}+k_{2}}{k_{2}}\left(y-y_{4}-y_{4}\ln\frac{y}{y_{4}}\right)+\frac{d_{3}\left(d_{2}+k_{2}\right)+\left(d_{2}+k_{2}\right)pz_{4}}{ak_{2}}\\ \times \left(v-v_{4}-v_{4}\ln\frac{v}{v_{4}}\right)+\frac{d_{3}\left(d_{2}+k_{2}\right)+\left(d_{2}+k_{2}\right)pz_{4}}{ak_{2}}\frac{q}{g}\left(w-w_{4}-w_{4}\ln\frac{w}{w_{4}}\right)\\ +\frac{p}{c}\frac{d_{2}+k_{2}}{k_{2}}\left(z-z_{4}-z_{4}\ln\frac{z}{z_{4}}\right). \end{array}$$

Then, we have

(53) $$\begin{array}{c} \dot{ℒ}\left(x,s,y,v,w,z\right)=\lambda -d_{1}x-\left(d_{2}+k_{2}\right)s_{4}\frac{x+v_{4}}{k_{1}xv_{4}}\left(\lambda -d_{1}x-\frac{k_{1}xv}{x+v}\right)-\frac{s_{4}}{s}\frac{k_{1}xv}{x+v}+\left(d_{2}+k_{2}\right)s_{4}\\ +\frac{d_{3}\left(d_{2}+k_{2}\right)}{k_{2}}y_{4}-\left(d_{2}+k_{2}\right)\frac{y_{4}}{y}s-\frac{d_{3}d_{4}\left(d_{2}+k_{2}\right)+d_{4}\left(d_{2}+k_{2}\right)pz_{4}}{ak_{2}}v\\ -\frac{d_{3}\left(d_{2}+k_{2}\right)+\left(d_{2}+k_{2}\right)pz_{4}}{k_{2}}\frac{v_{4}}{v}y+\frac{d_{3}d_{4}\left(d_{2}+k_{2}\right)+d_{4}\left(d_{2}+k_{2}\right)pz_{4}}{ak_{2}}v_{4}\\ -\frac{d_{3}\left(d_{2}+k_{2}\right)+\left(d_{2}+k_{2}\right)pz_{4}}{ak_{2}}qw_{4}v+\frac{d_{3}\left(d_{2}+k_{2}\right)+\left(d_{2}+k_{2}\right)pz_{4}}{ak_{2}}qv_{4}w_{4}\\ +\frac{\left(d_{2}+k_{2}\right)}{k_{2}}pz_{4}y_{4}. \end{array}$$

We know that

(54) $$\begin{array}{c} \left\{\begin{array}{c} \lambda =d_{1}x_{4}+\left(d_{2}+k_{2}\right)s_{4},\\ k_{1}x_{4}v_{4}=\left(x_{4}+v_{4}\right)\left(d_{2}+k_{2}\right)s_{4},\\ \frac{s_{4}}{y_{4}}=\frac{d_{3}}{k_{2}}+\frac{pz_{4}}{k_{2}},\\ \frac{y_{4}}{v_{4}}=\frac{d_{4}}{a}+\frac{qw_{4}}{a},\\ \frac{s_{4}}{v_{4}}=\frac{d_{3}d_{4}}{ak_{2}}+\frac{d_{4}pz_{2}}{ak_{2}}+\frac{d_{3}qw_{4}}{ak_{2}}+\frac{pqz_{4}w_{4}}{ak_{2}}, \end{array}\right. \end{array}$$

then,

(55) $$\begin{array}{c} \left\{\begin{array}{c} \lambda -d_{1}x=\left(d_{1}x_{4}+d_{2}+k_{2}\right)s_{4}-d_{1}x,\\ \lambda -d_{1}x-\left(d_{2}+k_{2}\right)s_{4}\frac{x+v_{4}}{k_{1}xv_{4}}\left(\lambda -d_{1}x-\frac{k_{1}xv}{x+v}\right)=d_{1}x_{4}\left(1-\frac{x}{x_{4}}-\frac{x_{4}}{x}\frac{x+v_{4}}{x_{4}+v_{4}}+\frac{x+v_{4}}{x_{4}+v4}\right)\\ +\left(d_{2}+k_{2}\right)s_{4}\left(1-\frac{x_{4}}{x}\frac{x+v_{4}}{x_{4}+v_{4}}+\frac{v}{v_{4}}\frac{x+v_{4}}{x+v}\right)\\ -\frac{s_{4}}{s}\left(\frac{k_{1}xv}{x+v}\right)+\left(d_{2}+k_{2}\right)s_{4}=\left(d_{2}+k_{2}\right)s_{4}\left(1-\frac{s_{4}}{s}\frac{xv}{x_{4}v_{4}}\frac{x_{4}+v_{4}}{x+v}\right), \end{array}\right.\\ \left\{\begin{array}{c} \frac{d_{3}\left(d_{2}+k_{2}\right)}{k_{2}}y_{4}+\frac{d_{2}+k_{2}}{k_{2}}py_{4}z_{4}-\left(d_{2}+k_{2}\right)\frac{y_{4}}{y}s=\left(d_{2}+k_{2}\right)s_{4}\left(1-\frac{y_{4}}{y}\frac{s}{s_{4}}\right),\\ -\frac{d_{3}d_{4}\left(d_{2}+k_{2}\right)+d_{4}\left(d_{2}+k_{2}\right)pz_{4}}{ak_{2}}v-\frac{d_{3}\left(d_{2}+k_{2}\right)+\left(d_{2}+k_{2}\right)pz_{4}}{k_{2}}\frac{v_{4}}{v}y\\ +\frac{d_{3}d_{4}\left(d_{2}+k_{2}\right)+d_{4}\left(d_{2}+k_{2}\right)pz_{4}}{ak_{2}}v_{4}-\frac{d_{3}\left(d_{2}+k_{2}\right)+\left(d_{2}+k_{2}\right)pz_{4}}{ak_{2}}qw_{4}v\\ +\frac{d_{3}\left(d_{2}+k_{2}\right)+\left(d_{2}+k_{2}\right)pz_{4}}{ak_{2}}qv_{4}w_{4}\\ =\left(d_{2}+k_{2}\right)s_{4}\left(1-\frac{v_{4}}{v}\frac{y}{y_{4}}-\frac{v}{v_{4}}\right). \end{array}\right. \end{array}$$

This fact implies that

(56) $$\begin{array}{c} \dot{ℒ}\left(x,s,y,v,w,z\right)=-\frac{d_{1}v_{4}}{x\left(x_{4}+v_{4}\right)}{\left(x-x_{4}\right)}^{2}\\ -\left(d_{2}+k_{2}\right)s_{4}\left(\frac{x{\left(v-v_{4}\right)}^{2}}{v_{4}\left(x+v_{4}\right)\left(x+v\right)}\right)\\ +\left(d_{2}+k_{2}\right)s_{4}\left(5-\frac{x_{4}}{x}\frac{x+v_{4}}{x_{4}+v_{4}}-\frac{s_{4}}{s}\frac{xv}{x_{4}v_{4}}\frac{x_{4}+v_{4}}{x+v}-\frac{sy_{4}}{s_{4}y}-\frac{yv_{4}}{y_{4}v}-\frac{x+v}{x+v_{4}}\right). \end{array}$$

Since the arithmetic mean is greater than or equal to the geometric mean, it follows that

(57) $$\begin{array}{c} 5-\frac{x_{4}}{x}\frac{x+v_{4}}{x_{4}+v_{4}}-\frac{s_{4}}{s}\frac{xv}{x_{4}v_{4}}\frac{x_{4}+v_{4}}{x+v}-\frac{sy_{4}}{s_{4}y}-\frac{yv_{4}}{y_{4}v}-\frac{x+v}{x+v_{4}}\leq 0, \end{array}$$

which means that $\dot{ℒ}\leq 0$, and the equality holds when *x*=*x*_4_, *s*=*s*_4_, *y*=*y*_4_, *v*=*v*_4_, *w*=*w*_4_, and *z*=*z*_4_. By the LaSalle invariance principle [14], the endemic point *E*_4_ is globally asymptotically stable when *R*_0_ > 1.

## 3. Numerical Results

**Figure 2:**
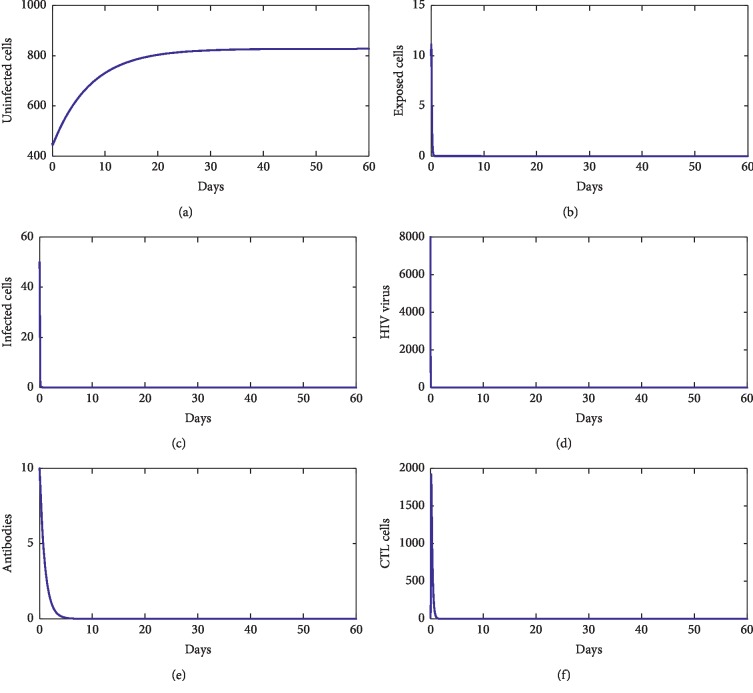
The behavior of the disease for *λ*=10, *d*_1_=0.0139, *k*_1_=0.04, *d*_2_=0.0495, *k*_2_=1.1, *d*_3_=0.5776, *a*=2, *d*_4_=0.6, *q*=0.05, *g*=10^−11^, *h*=0.1, *p*=0.0024, *c*=0.15, and *b*=0.5.

**Figure 3:**
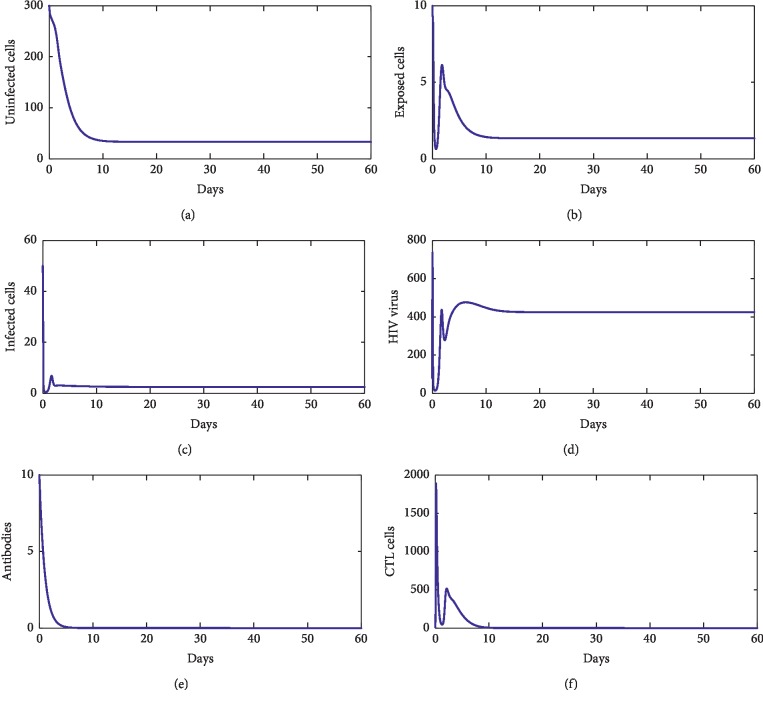
The behavior of the disease for *λ*=2, *d*_1_=0.0139, *k*_1_=0.05, *d*_2_=0.0495, *k*_2_=1.1, *d*_3_=0.5776, *a*=100, *d*_4_=0.6, *q*=0.05, *g*=10^−11^, *h*=0.1, *p*=0.0024, *c*=0.15, and *b*=0.5.

**Figure 4:**
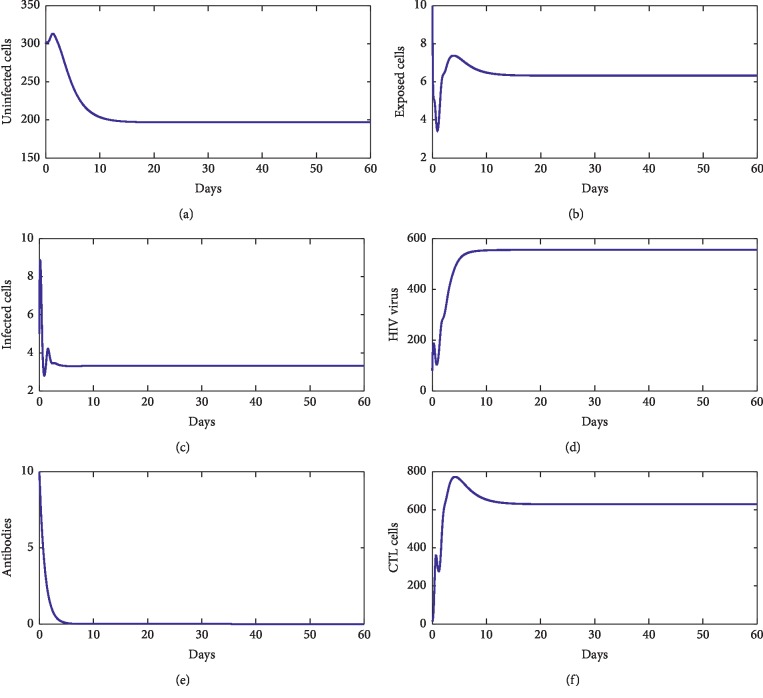
The behavior of the disease for *λ*=10, *d*_1_=0.0139, *k*_1_=0.05, *d*_2_=0.0495, *k*_2_=1.1, *d*_3_=0.5776, *a*=100, *d*_4_=0.6, *q*=0.05, *g*=10^−11^, *h*=0.1, *p*=0.0024, *c*=0.15, and *b*=0.5.

**Figure 5:**
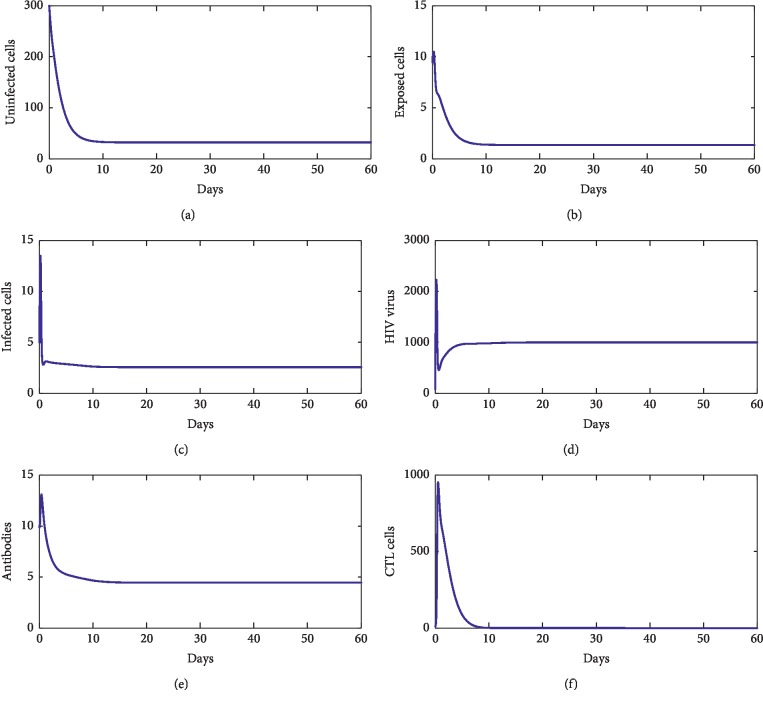
The behavior of the disease for *λ*=2, *d*_1_=0.0139, *k*_1_=0.05, *d*_2_=0.0495, *k*_2_=1.1, *d*_3_=0.5776, *a*=500, *d*_4_=0.6, *q*=0.05, *g*=10^−4^, *h*=0.1, *p*=0.0024, *c*=0.15, and *b*=0.5.

**Figure 6:**
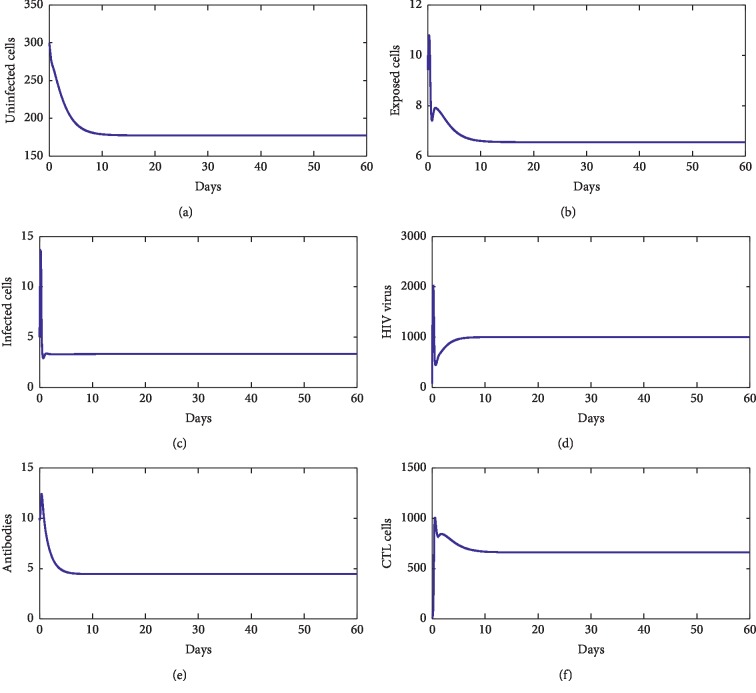
The behavior of the disease for *λ*=10, *d*_1_=0.0139, *k*_1_=0.05, *d*_2_=0.0495, *k*_2_=1.1, *d*_3_=0.5776, *a*=800, *d*_4_=0.6, *q*=0.5, *g*=10^−4^, *h*=0.1, *p*=0.0024, *c*=0.15, and *b*=0.5.

For our numerical simulations, system (1) is solved using the Runge–Kutta method iterative scheme. The numerical ranges of our parameters are given in Table 1.  Figure 2 shows the behavior of disease during the first 60 days of observation. From this figure, we observe that the solution converges to the point *E*_*f*_=(827.22, 0,0,0,0,0). With these chosen parameters, we have *R*_0_=0.22 < 1, which proves that *E*_*f*_ is stable. This supports our theoretical findings.  Figure 3 shows the behavior of the disease during 60 first days. From this figure, we observe that the solution of (1) converges towards the point *E*_1_=(33.17, 1.33, 2.54, 4.24 × 10^2^, 0,0). With these chosen parameters, we have *R*_0_=13.81 > 1, *R*_1_^*z*^=7.64 × 10^−1^ < 1, and *R*_1_^*w*^=4.24 × 10^−8^ < 1. This fact supports that *E*_1_ is stable.  Figure 4 shows the behavior of disease during 60 days. We observe that the solution of (1) converges towards the endemic point *E*_2_=(1.96 × 10^2^, 6.32, 3.33, 5.55 × 10^2^, 0,6.28 × 10^2^). In this figure, we have *R*_0_=13.81 > 1, *R*_1_^*z*^=3.81 > 1, and *R*_2_^*w*^=5.55 × 10^−8^ < 1, which supports the fact that *E*_2_ is stable.  Figure 5 shows the behavior of disease during the first 60 days of observation. We clearly see that the solution of (1) converges towards the endemic point *E*_3_=(32.07, 1.35, 2.57, 1000,4.45, 0). With the chosen parameters, we have *R*_0_=2.39 × 10^2^ > 1, *R*_3_^*z*^=0.77 < 1, and *R*_1_^*w*^=9.61 > 1; this supports the stability of *E*_3_. In addition, Figure 6 shows the behavior of disease for the first 60 days. We remark that the solution converges towards the last endemic point *E*_4_=(1.77 × 10^2^, 6.55, 3.33, 1000,4.46, 6.61 × 10^2^). With the used parameters, we have *R*_0_=75.31 > 1, *R*_3_^*z*^=3.74 > 1, and *R*_2_^*w*^=3.03 > 1; this confirms the theoretical result concerning the stability of *E*_4_.

### 3.1. Comparison with the Clinical Data

First, define the following objective function:

(58) $$\begin{array}{c} J=\frac{1}{n}\overset{n}{\underset{i=1}{\sum }}\log\;{\left(v\left(t_{i}\right)-\log\left(\hat{v}\left(t_{i}\right)\right)\right)}^{2}, \end{array}$$

where *v*(*t*_*i*_) represents the virus concentration at time *t*_*i*_ using the mathematical model (1) and $\hat{v}\left(t_{i}\right)$ represents the virus concentration clinical data at time *t*_*i*_ [18].

The numerical simulations are performed and compared to three patients' data picked from [18]. The data were from the University of Washington study [7] and from the Aaron Diamond AIDS Research Center (see Table 2).

**Figure 7:**
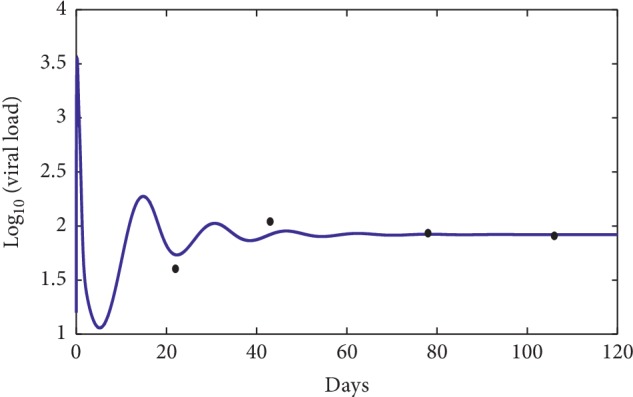
Comparison between the mathematical model (solid line) and the clinical data of the first patient [18] (dots). The used parameters for the model are *λ*=10, *d*_1_=0.0139, *k*_1_=0.05, *d*_2_=0.0495, *k*_2_=1.1, *d*_3_=0.5776, *a*=850, *d*_4_=0.6, *q*=0.5, *g*=1.2 × 10^−3^, *h*=0.1, *p*=0.0024, *c*=0.15, and *b*=0.5.

**Figure 8:**
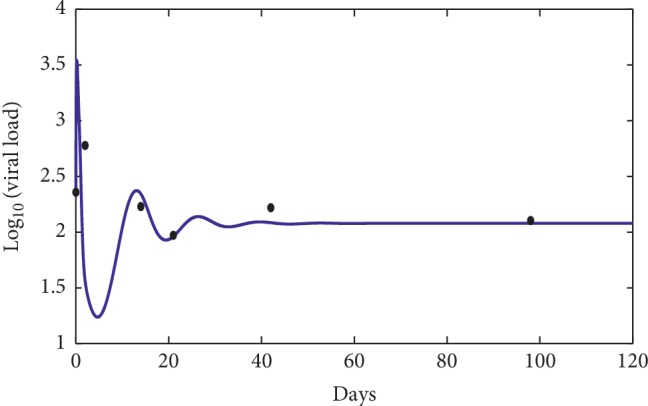
Comparison between the mathematical model (solid line) and the clinical data of the fifth patient [18] (dots). The used parameters for the model are *λ*=10, *d*_1_=0.0139, *k*_1_=0.05, *d*_2_=0.0495, *k*_2_=1.1, *d*_3_=0.5776, *a*=650, *d*_4_=0.6, *q*=0.5, *g*=10^−3^, *h*=0.12, *p*=0.0024, *c*=0.15, and *b*=0.5.

**Figure 9:**
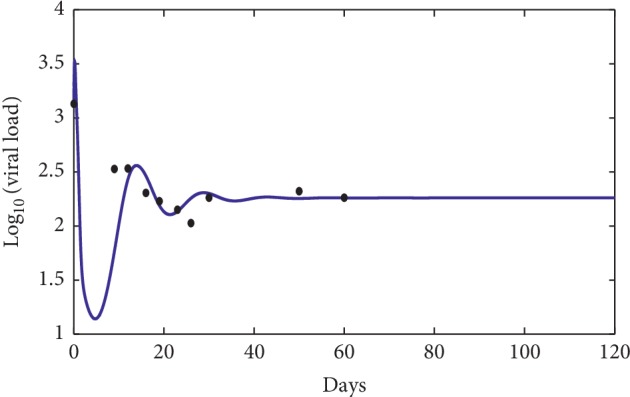
Comparison between the mathematical model (solid line) and the clinical data of the seventh patient [18] (dots). The used parameters for the model are *λ*=10, *d*_1_=0.0139, *k*_1_=0.05, *d*_2_=0.0495, *k*_2_=1.1, *d*_3_=0.5776, *a*=600, *d*_4_=0.6, *q*=0.5, *g*=10^−3^, *h*=0.182, *p*=0.0024, *c*=0.15, and *b*=0.5.

In Figure 7, the dots show the evolution of the infection during the first 120 days for the first patient [18], while the solid curve represents the numerical simulation of our suggested model. The error between the numerical simulation and the clinical data is approximately *J* ≈ 2.378 × 10^−1^ which indicates that the numerical simulation is a good approximation of the clinical data. Figures 8 and 9 show a comparison between the clinical data (dots) and the mathematical model (solid line), and the error is approximately *J* ≈ 8.43 × 10^−2^ and *J* ≈ 1.64 × 10^−1^, respectively. These three results indicate that our mathematical model can fit the clinical data of different patients for the first days of observations. However, the limit of our model is to predict a long time behavior of the infection disease.

### 3.2. Sensitivity Analysis

Using the method outlined in [19], we perform a sensitivity analysis using partial rank correlation coefficients (PRCC) to identify the main drivers of the basic reproduction number *R*_0_. Parameters were tested within the ranges given in Table 1.

**Figure 10:**
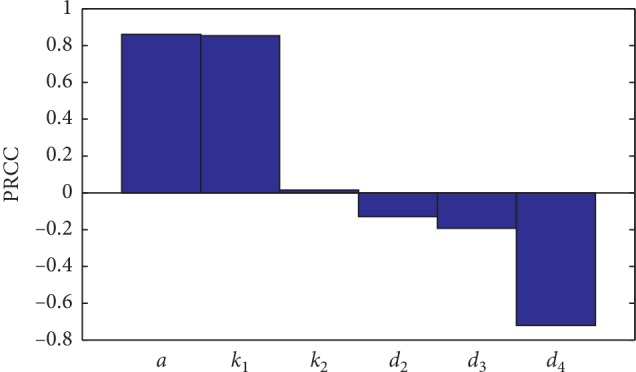
Sensitivity analysis of *R*_0_ to different input parameters of the model.

In Figure 10, we observe that *a* and *k*_1_ is highly positively correlated with *R*_0_. However, *d*_4_ has a strong negative correlation with *R*_0_. The other parameters *k*_2_, *d*_2_, and *d*_3_ present a weak correlation with *R*_0_. From the biological point of view, the sensitivity analysis shows that an increase of production rate of the virus by infected cells *a* or an increase of the infection rate *k*_1_ leads to an increase of the basic reproduction number *R*_0_. However, an increase in the clearance rate of virus *d*_4_ leads to a significant decease of the basic reproduction number *R*_0_.

## 4. Conclusion

In this paper, we have presented and studied a mathematical model describing HIV viral infection with saturated rate in the presence of the adaptive immune response. This adaptive immunity is represented by CTL immune response and antibodies. By using suitable Lyapunov functionals, the global stability of each equilibrium has been established. More precisely, the disease-free equilibrium is globally asymptotically stable when the basic reproduction number is below unity (*R*_0_ ≤ 1). Also, the endemic steady state *E*_1_ is globally asymptotically stable when *R*_0_ ≥ 1, *R*_1_^*z*^ ≤ 1, and *R*_1_^*w*^ ≤ 1. In presence of the adaptive immune response governed by competition between CTL and antibody responses, system (1) admits three infection steady states. The first infection steady state *E*_2_ is with only the presence of CTL response which is globally asymptotically stable if *R*_1_^*z*^ ≥ 1 and *R*_2_^*w*^ ≤ 1. The second infection steady state *E*_3_ is with only the presence of the antibody response which is globally asymptotically stable if *R*_1_^*w*^ ≥ 1 and *R*_3_^*z*^ ≤ 1. The third infection steady state is *E*_4_ with the activation of the antibodies and the CTL response at the same time. In this case, this equilibrium *E*_4_ is globally asymptotically stable when *R*_2_^*w*^ ≥ 1 and *R*_3_^*z*^ ≥ 1. In addition, different numerical simulations are performed in order to confirm the theoretical findings and to show that the adaptive immune response is responsible to reduce the viral load, increase the uninfected cells, and decrease the infected cells. Moreover, a comparison with some clinical data shows that our suggested model can be considered as a good approximation of the clinical tests especially for the first days of observation.

## Figures and Tables

**Table 1:** Parameters and their symbols and default values used in the suggested HIV model.

Parameters	Units	Meaning	Value	References
*Λ*	cells *μ*l^−1^ day^−1^	Source rate of CD4^+^ T cells	[0,10]	[15]
*k*_1_	*μ*l virion^−1^ day^−1^	Average of infection	[2.5 × 10^−4^, 0.5]	[9]
*d*_1_	day^−1^	Decay rate of healthy cells	0.0139	[9]
*d*_2_	day^−1^	Death rate of exposed CD4^+^ T cells	0.0495	[9]
*k*_2_	day^−1^	The rate that exposed cells become infected CD4^+^ T cells	1.1	[9]
*d*_3_	day^−1^	Death rate of infected CD4^+^ T cells, not by CTL killing	0.5776	[9]
*a*	day^−1^	The rate of production the virus by infected CD4^+^ T cells	[2,1250]	[9]
*d*_4_	day^−1^	Clearance rate of virus	[0.3466, 2.4]	[9]
*Q*	*μ*l virion days^−1^	Killing rate of antibody	0.5	[16]
*G*	*μ*l virion days^−1^	Activation rate CTL cells	10^−11^, 10^−4^	[16]
*H*	day^−1^	Death rate of antibody	0.1	[16]
*P*	*μ*l cell^−1^day^−1^	Clearance rate of infection	0.0024	[17]
*C*	cells cell^−1^ day^−1^	Activation rate CTL cells	0.15	[17]
*b*	day^−1^	Death rate of CTL cells	0.5	[17]

**Table 2:** The used clinical data [18] for Figure 7 (A), for Figure 8 (B), and for Figure 9 (C).

Clinical day test	Viral load (virions per *μ*l)
*A*	
22	27.7
43	210
78	85.9
106	81.1

*B*	
0	228.8
2	599.2
14	169.6
21	93.7
42	165.6
98	127

*C*	
0	1350.6
9	337.2
12	340.6
16	202.3
19	169.7
23	141.4
26	56.48
30	182.75
50	267
60	182.7

## Data Availability

All the used data for our simulations are cited in the manuscript and can be founded in the references.
